# Engenolacetic Acid

**Published:** 1893-02-11

**Authors:** 


### Contemporary Theory and Research.

### ENGENOLACETIC ACID.

This is a new anæsthetic for the preparation of which the Maison Meister, Lucius, and Bruning have taken an award. Applied on the tongue as a fine powder it produces in the part with which it comes in contact complete insensibility, without irritation, for a longer or shorter time. This substance appears under the aspect of brilliant flakes when it is crystallised in water, or as fine needles when crystallised in any alcoholic solution. It melts at 110°, and is obtained by submitting the ethylic ether of engenolacetic acid to the action of an alcoholic solution of ammonia. *(Journal de Medecine de Paris.)*

